# The efficacy of administration of prazosin in reducing the risk of recurrent urethral obstruction in male cats

**DOI:** 10.18849/ve.v8i4.638

**Published:** 2023-11-01

**Authors:** Rebecca Hearne+

**Keywords:** CATS, FELINE, PRAZOSIN, RECURRENT URETHRAL OBSTRUCTION

## Abstract

**PICO question:**

In male cats presenting with urethral obstruction, does administration of prazosin compared with no administration of prazosin reduce the incidence of recurrent urethral obstruction within the first month of initial presentation?

**Clinical bottom line:**

**Category of research:**

Treatment.

**Number and type of study designs reviewed:**

A total of three studies were included in this appraisal. Two of which were prospective double-blind clinical studies and one retrospective study.

**Strength of evidence:**

Weak.

**Outcomes reported:**

Both double-blind studies found no significant difference between prazosin administration and the development of recurrent urethral obstruction (rUO) when compared with a placebo. Both studies, however, did report that cats had a shorter urinary catheterisation time with prazosin administration. The retrospective study found no association between prazosin administration and the risk of rUO and found that after 14 days post discharge, significantly more 73/302 (24%) cats that received prazosin had experienced rUO compared to 11/86 (13%) of cats that did not. One study found side effects to the administration of prazosin which may be detrimental to the overall recovery.

**Conclusion:**

Administration of prazosin does not reduce the risk of rUO within 30 days of presentation. However, the strength of evidence is weak and would benefit from further clinical studies.

## How to apply this evidence in practice

The application of evidence into practice should take into account multiple factors, not limited to: individual clinical expertise, patient’s circumstances and owners’ values, country, location or clinic where you work, the individual case in front of you, the availability of therapies and resources.

Knowledge Summaries are a resource to help reinforce or inform decision making. They do not override the responsibility or judgement of the practitioner to do what is best for the animal in their care.

## The evidence

One paper compared prazosin against another drug, phenyoxybenazamine (Hetrick & Davidow, 2013), however, until 2017 there were no papers that analysed the effectiveness of prazosin compared to a placebo or no drug administration.

Three papers were found which were relevant to the PICO question (Reineke et al., 2017; Hanson et al., 2021; and Conway et al., 2022). In terms of study design the two double-blind studies are much higher on the hierarchy of evidence compared to the retrospective study. However, they did not have adequate numbers of participants to draw statistically reliable conclusions. Because of this, the overall strength of evidence is weak.

## Summary of the evidence

**Reineke et al. (2017):** 

Population:	A population of male cats presented to a university teaching hospital following urethral obstruction. Exclusion criteria: owner unable to give medication; presence of urinary calculi identified on ultrasound; chronic kidney or heart disease (or prior administration of vasoactive therapies); cats already on treatment for urethral obstruction; or with an indwelling urinary catheter in place on presentation.
Sample size:	72 male cats were enrolled onto the study of these, 44 male cats completed the study.
Intervention details:	All cats were stabilised before any intervention. Once cardiovascularly stable cats were sedated with 0.1–0.4 mg/kg methadone given via intravenous route (IV) and 0.1–0.4 mg/kg midazolam IV, plus or minus incremental amounts of propofol IV as needed throughout the procedure. Sterile lidocaine gel was applied to the tip of the penis. A urinary catheter was placed using a standardised protocol. The bladder was emptied and flushed until urine appeared grossly clear. The urinary catheter was secured in place and attached to a closed collection urinary drainage system. Cats were randomised in a double-blinded fashion to receive either prazosin (0.25 mg/cat PO q12 hours) or a placebo in an identical gelatin capsule (1 x capsule PO q12 hours) for 30 days starting as soon as the cat was able to receive oral medication following the relief of the blockage. Twenty cats received the placebo and 27 received prazosin. There was a weekly follow-up for the first 30 days which included asking about evidence of lower urinary tract signs using a Likert scale and occurrence of re-obstruction. They also asked about any potential adverse effects.
Study design:	Double-blinded, prospective, interventional study.
Outcome studied:	The rate of recurrent urethral obstruction (rUO) amongst the two treatment groups. This was measured by a weekly follow-up to 30 days post initial blockage.
Main Findings (relevant to PICO question)	There was no significant difference in the rUO rates between the two treatment groups. There was also no difference in the reporting of lower urinary tract signs reported by the owner between the placebo and treatment groups 1 month following discharge (P = 0.776).
Limitations:	Mixed population of different breeds and neuter status. Some cats had a history of urethral blockage. Cats spent a variable amount of time in hospital and with a urinary catheter. Heavily reliant on the owner compliance following discharge instructions. There is a risk of type I and II statistical errors due to the small sample size. This study did not exclusively include cats that were presenting for the first time and so may have pre-existing urinary pathology which would contribute to the rate of re-blocking.

**Hanson et al. (2021):** 

Population:	A population of castrated male cats presenting to the hospital for the first time with urethral obstruction from 2014 to August 2017. Exclusion criteria: animals that were on medication or had a urinary catheter passed on presentation. Also excluded were animals with concurrent disease, for example heart disease or hypertension.
Sample size:	80 cats were enrolled and placed into randomised groups, treatment, or control (40 participants in each group).
Intervention details:	This was a double-blind study. All cats were treated with a standard anaesthetic and analgesic protocol. An indwelling urinary catheter was placed, and bladder flushed in a standardised protocol. The cats were then attached to a closed urinary collection system and hospitalised for care. Fifteen cats were excluded after the study start date and therefore did not complete the study. The reason for exclusion was largely due to animals not receiving a full 7 day course of medication (12 cats). In addition, one cat from the placebo group was withdrawn by the owner and two further cats from this group lost to follow-up. A total of 65 cats completed the study. The cats were randomly split into two treatment groups. Of the 65 cats that completed the study, 37 were in the group treated with prazosin (0.5 mg/cat PO q12 hours for 7 days). and 28 in the group treated with placebo (1 x capsule PO q12 hours for 7 days). A follow-up via telephone after 30 days was conducted for all animals to identify which of the cats had experienced further urethral obstruction.
Study design:	Randomised prospective interventional study.
Outcome studied:	The rates of cats who developed recurrent urethral obstruction (rUO) following treatment with prazosin versus those given the placebo. This was measured by a follow-up phone call to assess the rates of rUO.
Main Findings (relevant to PICO question)	37/40 cats from the prazosin group completed the study. 28/40 cats from the placebo groups completed the study. 16/65 (25%) cats experienced rUO within the 30 days following the initial blockage: Of these 16 cats, five cats were in the placebo group, and 11 cats were in the prazosin group. 10/16 cats re-blocked whilst still hospitalised. There was no significant difference in the rate of re-blocking for the cat’s receiving prazosin and those that were receiving the placebo (P = 0.27).
Limitations:	This study was reliant on owner compliance, for example: owners giving the medication exactly as prescribed and there is no way to ensure this is happening. Small sample size. There are many variables within the individuals environment, which may contribute to rUO, such as diet, access to litter trays and water. Radiography was used to check for cystoliths which is less sensitive than ultrasonography.

**Conway et al. (2022):** 

Population:	Multicentre population of male cats with urethral obstruction that were treated by veterinarians in the USA. Exclusion criteria for cats: urolithiasis, development of a urethral tear, urinary tract neoplasia, or insufficient detail on the medical record.
Sample size:	485 cats enrolled in the study.
Intervention details:	There were two parts to this study. Part 1: Cats were split into two groups. The key variable was whether the cat received prazosin (intervention) n = 302 or did not receive prazosin as part of the treatment for obstruction (comparison) n = 86. The intervention group received 0.5 to 1 mg of prazosin, PO, once daily for 14 days. The comparison group received nothing. Additional information about the cats with urethral obstruction was used to conduct the Fisher extract test on further variables including the method and ease of passing a urinary catheter, clarity of the urine at the time of unblocking and cat characteristics and to determine whether these variables impacted the choice of medication. Part 2: The second part of this study involved combining raw data from previous primary studies (Hansen et al., 2021; Hetrick & Davidow, 2013; and Reineke et al., 2017) to further evaluate the impact of prazosin on rates of recurrent urethral obstruction (rUO). A total of 700 cats with urethral obstruction (UO) were evaluated. 128/700 (18.3%) had experienced rUO, the time scale of which is not described here. Of the 700 cats, 505 received a treatment of prazosin (varying dosage rates) and 195 did not receive prazosin. Part two does not relate to the PICO question and therefore will not be commented on further in this Knowledge Summary.
Study design:	Retrospective cohort study.
Outcome studied:	Rates of recurrent urethral obstruction in the two groups (treatment and comparison). This was measured by comparing the rates of development of rUO between the two groups prior to hospital discharge and by day 14. There were additional relevant outcomes studied; such as the characteristic nature of the blockage (e.g. gritty feel within the urethra) and whether this had any links with the rates of rUO in cats that did and did not receive prazosin as part of treatment.
Main Findings (relevant to PICO question)	485 cats started the trail however, 97 cats were removed due to the presence of urolithiasis, urethral tears, or incomplete medical records. 388 male cats completed the study. Within 14 days following discharge a higher proportion of the cats treated with prazosin experienced rUO compared to those not treated with prazosin: 73/302 (24%) of cats from the prazosin treated group developed rUO 34/73 cats (34/30 [11.3%] of the total number) developed rUO whilst still hospitalised. 11/86 (13%) of non-prazosin treated cats developed rUO 5/11 cats (5/86 [5.8%] of the total number) developed rUO whilst still hospitalised. Prior to discharge there was no association with administration of prazosin and risk of rUO. In addition, it was noted that the presence of crystalluria (P = 0.40), difficulty of catheterisation (P = 0.01) or a gritty feeling when passing urinary catheter (P = 0.01) were all associated with an increased risk of rUO.
Limitations:	Due to the nature of the design of the study, there is likely to be a selection bias (for example clinicians only remembering to report cases that have been administered prazosin) and thus may not be able to be generalised. This is demonstrated by the treatment group making up 302/388 (77.%) of all cases reported and the cats not treated with prazosin only made up 86/388 (22%). The difference in number of cats between these groups may have reduced the validity of the study. The design of the study means that it is difficult to control variables. No standardised treatment protocols were able to be followed.

## Appraisal, application and reflection

The goal of this Knowledge Summary was to ascertain as to whether treatment with prazosin after urethral obstruction in cats, is beneficial in preventing recurrent urethral obstruction (rUO). It is hypothesised that urethral spasm is a potential cause of rUO, therefore administration of prazosin, a smooth muscle relaxant, was thought to help prevent rUO (Straeter-Knowlen et al., 1995).

The available evidence fitting to the PICO question comes from three published papers (Reineke et al., 2017; Hanson et al., 2021; and Conway et al., 2022). One study is a double-blind prospective interventional study (Reineke et al., 2017), and this study type produces the highest level of evidence as there is minimal bias from the client and the researcher. However, a downside to this study is the lack of study participants. Only 47 cats took part in this study (27 in the treatment group and 20 in the placebo group). Calculations carried out after the completion of the study suggest that a total of 1,915 cats (1149 in the prazosin-treated group and 766 in the placebo-treated group) would have been needed for an effect to be identified (Reineke et al., 2017). This lack of participants makes it difficult to generalise to the whole population. Similarly, the Hanson et al. (2021) study is a randomised double-blind study, so again ranks high on the hierarchy of evidence, however, there is a risk of type I and II statistical errors due to the small sample size and so potential lack of generalisable data. Hanson et al. (2021) states that to truly determine the statistical difference 199 study participants would be needed, as opposed to the actual study size of 65/80 cats that completed the study.

The third study is lower on the hierarchy of evidence and is a retrospective, observational cohort study (Conway et al., 2022), this means that drawing concrete conclusions from this third study is challenging as there are so many variables and areas for bias, compared to double-blinded prospective studies. For example, all cats were treated by different veterinarians at different hospitals and the collection of data is relying on the clinical notes for each animal.

Following discharge from the hospital it is very difficult to control variables within the cat’s environment which may contribute to the development of rUO. Reineke et al. (2017) made an attempt to overcome this with standardised discharge instructions for each patient which included elements such as litter tray placement, hygiene, and increasing the cats water consumption. However, it is impossible to control these variables completely. An additional variable was that there was a range of doses of prazosin used across each study (varying from 0.25 mg/cat q12hrs, 0.5 mg/cat q12hrs or 1 mg/cat per os once daily), however, despite this, similar conclusions were reached (Reineke et al., 2017; Hanson et al., 2021; and Conway et al., 2022).

Study populations were all made up of male cats presenting with urethral obstruction. There were limited exclusion criteria based on demographics (breed, age, or neutered status) in all three studies. However, two studies (Reineke et al., 2017; and Hanson et al., 2021) excluded cats with underlying diseases such as chronic kidney disease or hypertension which the cat may have been on concurrent medication for. Due to the Conway et al., (2022) study being a retrospective study there is no note of such exclusion criteria. All studies excluded cats that experienced complications such as any ruptures or tears as well as those with suspected presence of cystoliths diagnosed via imaging. Hanson et al., (2021) was the only study which included cats presenting for the first time, meaning those cats have no prior history of urethral obstruction. Having a history of urethral obstruction may impact the results as it is possible there is some degree of penile or urethral trauma associated with prior urinary catheterisation (Corgozinho et al., 2007). In addition, all three studies excluded cats with urolithiasis or crystoliths as a possible cause of urethral obstruction. Ultrasound is the most sensitive method of detecting uroliths >2 mm (Reineke et al., 2017), however, there is no way to guarantee that cats did not have any urocystoliths <2 mm. Hanson et al. (2021), used radiography to detect the presence of urocystoliths, this is not a sensitive measurement (Kyles et al., 2005) and thus cats with urolithiasis as a cause of urethral obstruction may have been inadvertently included in the study.

It was reported that cats receiving prazosin had a shorter hospitalisation time and shorter duration of urinary catheterisation than those receiving the placebo (Reineke et al., 2017). Similarly, Hanson et al. (2021) noted that cats receiving prazosin had a shorter duration of urinary catheterisation compared to the group receiving the placebo. Although, there are many variables that could have led to a shorter duration of urinary catheterisation, such as owner finances or the self-removal of the urinary catheter by the patient it can be hypothesised that prazosin is potentially beneficial in the early stages of urethral obstruction. However, the limitation of the small sample size in both these studies precludes any firm conclusions being drawn and a similar study with a greater number of study participants would be needed to prove this hypothesis.

There is a previous retrospective study (Hetrick & Davidow, 2013) again low on the hierarchy of evidence, which concluded that cats had a lower risk of rUO when administered prazosin compared to phenyoxybenazamine. However, it was not possible to determine if this was because phenyoxybenazamine increases the risk of rUO as opposed to prazosin reducing the risk. Therefore, the three studies comparing prazosin to no drugs, or a placebo are a preferable source of evidence.

Overall, it can be concluded from all three studies that there is no benefit from the administration of prazosin post urethral obstruction towards preventing rUO, compared to no administration within 14 to 30 days after the initial obstruction. However, more clinical studies are needed with larger sample sizes. In addition, the side effects of giving prazosin may have detrimental effects on patient recovery from urethral obstruction. For example, Reineke et al. (2017) noted side effects such as lethargy, diarrhea, and anorexia, all of which may contribute to the development of dehydration, leading to worsening of kidney damage and clinical signs, especially if the cat is experiencing post unblocking diuresis.

## Methodology

**Search strategy:** 

Databases searched and dates covered:	CAB Abstracts on OVID Platform 1995–2023 PubMed 1995–2023
Search strategy:	CAB Abstracts: (Cat or feline) AND Urethral obstruction AND Prazosin PubMed: (cat or feline) AND (Prazosin or hypovase) AND “Urethral obstruction”
Dates searches performed:	21 Sep 2023

**Exclusion / inclusion criteria:** 

Exclusion:	Duplicate papers. Books. Case reports only involving a single animal. Articles not relevant to the PICO. Any papers not in English.
Inclusion:	Any primary research paper relevant to the PICO question.

**Search outcome:** 

Database	Number of results	Excluded –Not relevant to PICO	Excluded – No control group	Total relevant papers
CAB Abstracts	9	7	0	2
PubMed	9	5	1	3
Total relevant papers when duplicates removed				3

## ORCiD

Rebecca Hearne: https://orcid.org/0009-0003-4004-7161

## Conflict of interest

The author declares no conflicts of interest.

## Disclaimer

Knowledge Summaries are a peer-reviewed article type which aims to answer a clinical question based on the best available current evidence. It does not override the responsibility of the practitioner. Informed decisions should be made by considering such factors as individual clinical expertise and judgement along with patient’s circumstances and owners’ values. Knowledge Summaries are a resource to help inform and any opinions expressed within the Knowledge Summaries are the author’s own and do not necessarily reflect the view of the RCVS Knowledge. Authors are responsible for the accuracy of the content. While the Editor and Publisher believe that all content herein are in accord with current recommendations and practice at the time of publication, they accept no legal responsibility for any errors or omissions, and make no warranty, express or implied, with respect to material contained within. For further information please refer to our Terms of Use.
